# Genome-wide transcriptomic and phylogenetic analyses reveal distinct aluminum-tolerance mechanisms in the aluminum-accumulating species buckwheat (*Fagopyrum tataricum*)

**DOI:** 10.1186/s12870-014-0395-z

**Published:** 2015-01-21

**Authors:** Haifeng Zhu, Hua Wang, Yifang Zhu, Jianwen Zou, Fang-Jie Zhao, Chao-Feng Huang

**Affiliations:** State Key Laboratory of Crop Genetics and Germplasm Enhancement, College of Resources and Environmental Science, Nanjing Agricultural University, Nanjing, 210095 China; State Key Laboratory Breeding Base for Zhejiang Sustainable Pest and Disease Control, Institute of Crop and Nuclear Technology Utilization, Zhejiang Academy of Agricultural Sciences, Hangzhou, 310021 China

**Keywords:** Aluminum tolerance, Al-tolerance genes, Buckwheat, Homolog, Organic acid, Transcriptome

## Abstract

**Background:**

Similar to common buckwheat (*Fagopyrum esculentum*), tartary buckwheat (*Fagopyrum tataricum*) shows a high level of aluminum (Al) tolerance and accumulation. However, the molecular mechanisms for Al detoxification and accumulation are still poorly understood. To begin to elucidate the molecular basis of Al tolerance and accumulation, we used the Illumina high-throughput mRNA sequencing (RNA-seq) technology to conduct a genome-wide transcriptome analysis on both tip and basal segments of the roots exposed to Al.

**Results:**

By using the Trinity method for the *de novo* assembly and cap3 software to reduce the redundancy and chimeras of the transcripts, we constructed 39,815 transcripts with an average length of 1184 bp, among which 20,605 transcripts were annotated by BLAST searches in the NCBI non-redundant protein database. Gene Ontology (GO) and Kyoto Encyclopedia of Genes and Genomes (KEGG) enrichment analysis showed that expression of genes involved in the defense of cell wall toxicity and oxidative stress was preferentially induced by Al stress. Our RNA-seq data also revealed that organic acid metabolism was unlikely to be a rate-limiting step for the Al-induced secretion of organic acids in buckwheat. We identified two citrate transporter genes that were highly induced by Al and potentially involved in the release of citrate into the xylem. In addition, three of four conserved Al-tolerance genes were found to be duplicated in tartary buckwheat and display diverse expression patterns.

**Conclusions:**

Nearly 40,000 high quality transcript contigs were *de novo* assembled for tartary buckwheat, providing a reference platform for future research work in this plant species. Our differential expression and phylogenetic analysis revealed novel aspects of Al-tolerant mechanisms in buckwheat.

**Electronic supplementary material:**

The online version of this article (doi:10.1186/s12870-014-0395-z) contains supplementary material, which is available to authorized users.

## Background

Aluminum (Al) toxicity is a major limiting factor for crop production on acid soils, which make up over 30% of the world’s arable soils and up to 70% of the potential arable land [1]. On acidic soils with pH below 5.5, phytotoxic forms of Al (mainly Al^3+^) are solubilized into the soil solution, which inhibit root growth and thereafter limit water and mineral nutrient uptake, resulting in losses of crop yield [2]. To grow on Al-toxic environments, some plant species have evolved resistance mechanisms that enable them to tolerate toxic levels of Al.

Al-activated organic acid release from roots is a well-documented mechanism of Al detoxification [3,4]. Organic acids such as malate, citrate and oxalate are able to chelate Al and thereby attenuate Al toxicity. Different plants secrete different organic acids to detoxify Al. For example, wheat (*Triticum aestivum*), oilseed rape (*Brassica napus*) and *Arabidopsis thaliana* secrete malate after exposure to Al stress [5-7], while Al-tolerant cultivars of snapbean (*Phaseolus vulgaris*), rice bean (*Vigna umbellata*), maize (*Zea mays*), and soybean (*Glycine max*) release citrate in response to Al stress [8-12]. Oxalate can be secreted from the roots of buckwheat, tomato and spinach (*Spinacia oleracea*) upon exposure to Al stress [13-16]. Recently, genes responsible for the Al-activated secretion of malate and citrate have been identified. Sasaki *et al.* [17] cloned the first Al-resistant gene *ALMT1* in wheat, which encodes a plasma membrane transporter to transport malate from root cells to the rhizosphere for the chelation and detoxification of Al. Genes for citrate secretion were independently identified in barley [18] and sorghum [19], which were found to encode members of the multidrug and toxic compound extrusion (MATE) family. To date, genes involved in oxalate release have not been identified.

Using mutant screening and map-based gene cloning approaches on the model plants, rice and Arabidopsis, recent studies have unraveled some common Al-tolerant mechanisms in plants. ART1/STOP1 is a C2H2-type zinc-finger transcription factor, which is required for Al tolerance through regulation of downstream Al tolerance genes in both rice and Arabidopsis [20,21]. *STAR1* and *STAR2/ALS3* encode a nucleotide-binding domain and a transmembrane domain of an ABC (ATP-binding cassette) transporter, respectively. STAR1 and STAR2/ASL3 form a complex to transport UDP-glucose for the modification of cell walls thereby detoxifying Al [22-24]. ALS1 encodes a half-size ABC transporter and is involved in sequestering Al into the vacuoles for the internal detoxification of Al [25,26]. Although the functions of STAR1, STAR2/ALS3 and ALS1 in Al tolerance are conserved in plants, their expression patterns differ between rice and Arabidopsis. In general, the expression level and the level of induction by Al stress of these genes are higher in the Al-tolerant species rice than in the Al-sensitive species Arabidopsis, suggesting that Al-tolerant species may require increased expression of these conserved Al-tolerance genes to overcome Al stress.

Common buckwheat (*Fagopyrum esculentum*) is an Al-tolerant species and can accumulate Al to levels as high as 15,000 ppm in leaves, when grown on acid soils, without displaying symptoms of Al toxicity [27]. Physiological studies have demonstrated that common buckwheat secretes oxalate to detoxify Al externally and utilizes oxalate to chelate and sequester Al in the vacuoles of both roots and shoots for internal detoxification [13,14,28]. Although oxalate is required for Al translocation in buckwheat, Al in the xylem appears to be complexed with citrate instead of oxalate, suggesting that Al may undergo a ligand exchange from oxalate to citrate when Al is transported into the xylem [29]. However, understanding the molecular mechanisms of Al tolerance in buckwheat has been hampered by the lack of the genomic sequence and transcriptomic data under Al stress.

Recent advances in high-throughput mRNA sequencing (RNA-seq) offer the capability to discover new genes and transcripts and to quantify gene expression simultaneously. In the present study, we used the RNA-seq technique to analyze the transcriptome of different root zones of tartary buckwheat (*Fagopyrum tataricum*) in response to Al treatment. Tartary buckwheat was chosen in our study because it is an Al-accumulating species [30] but unlike common buckwheat, is self-pollinating, which makes it easier to assemble transcripts and to conduct further gene function analysis. We constructed high-quality genome-wide transcripts and examined the expression profile of Al-responsive genes in this buckwheat species. Combined with quantitative RT-PCR and phylogenetic analysis, our results revealed novel aspects of Al-tolerant mechanisms in tartary buckwheat.

## Results

### Al accumulation pattern in tartary buckwheat

**Figure 1:**
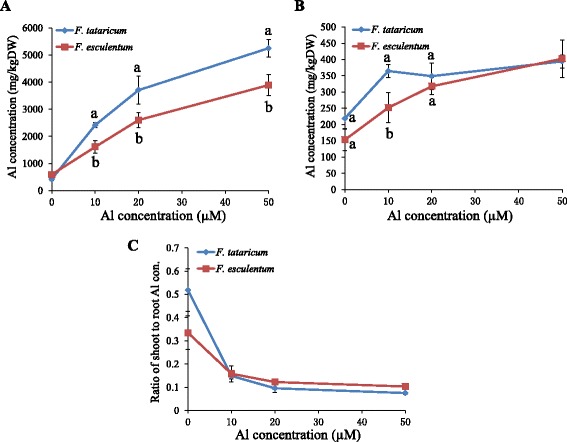
**Al accumulation in roots and shoots of** ***Fagopyrum tataricum*** **and** ***Fagopyrum esculentum*** **.** Two-week-old seedlings were exposed to a series of Al concentrations (0, 10, 20, 50 μM Al) for 8 d intermittently. The Al concentrations in roots **(A)** and shoots **(B)** and the ratio of shoot to root Al concentrations **(C)** were analyzed, respectively. Data are means ± SD (n = 4). Means with different letters are significantly different (P < 0.05, Tukey test).

To compare Al accumulation by tartary buckwheat and common buckwheat, we exposed plants of both species to different Al concentrations for 8 d intermittently in a hydroponic experiment. Both species accumulated appreciable amounts of Al in the roots and shoots in the control treatment (Figure 1A and B), suggesting that both buckwheat species efficiently took up the background level of Al in the nutrient solution. In the treatments with 10–50 μM Al, tartary buckwheat accumulated significantly more Al in the roots than common buckwheat (Figure 1A). Tartary buckwheat accumulated higher concentrations of Al in the shoots than common buckwheat in the 10 μM Al treatment, whereas shoot Al concentrations were similar between the two species in the higher Al treatments (20 and 50 μM) (Figure 1B).

In both species, the Al translocation efficiency from roots to shoots decreased with increasing Al concentration in the solution (Figure 1C). The shoot to root Al concentration ratio in tartary buckwheat decreased from 0.52 in the control treatment to 0.08 in the 50 μM Al treatment. This result suggests that xylem loading of Al might be the rate-limiting step controlling Al accumulation in the shoots in buckwheat.

### *De novo* assembly of the transcripts and annotation

**Figure 2:**
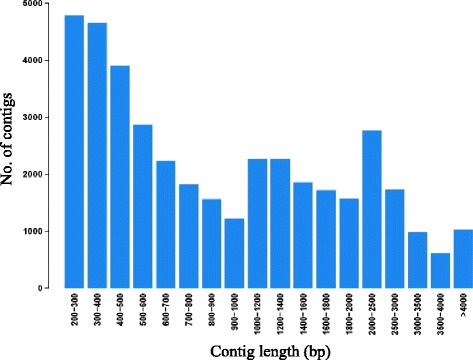
**Distribution of the length of transcript assembly contigs.**

For RNA-seq analysis, tartary buckwheat plants were treated with 50 μM Al for a short period of time (6 h). At this concentration root elongation was inhibited by 76% compared to the control (data not shown). Root tips and basal roots from both the control and + Al treatments were sampled for RNA isolation and Illumina paired-end RNA-seq. RNA-seq generated a range of 35.1 ~ 46.6 million clean reads on each sample (Additional file 1: Table S1). In total there were 267.4 million clean reads from all samples with an average length of 100 nucleotides per read and a GC content of 47.84% GC. These were used for the assembly of the transcripts. Because the genome sequence of buckwheat is not available, a *de novo* assembly method, the Trinity method [31,32], was used to construct the transcripts and 58,438 transcript contigs were assembled. In order to reduce the redundancy and chimeras of the transcripts, we used cap3 software to combine highly similar transcripts and retain the longest transcripts with the highest read coverage, and removed the transcripts with the lowest read coverage [31]. As a result, the number of contigs was further reduced to 39,815 (Additional file 2: Table S2). The assembled contigs had a length distribution from 201 to 25,284 bp, with an average length of 1184 bp (Figure 2). The average coverage for each assembled contig is 508 reads per base, indicating a high read coverage of the contigs.

Recently, Logacheva *et al.* [33] performed transcriptome sequencing in *F. tataricum* by 454 sequencing. In comparison with their results, we produced more assembled contigs (39,815 vs 25,401) and a longer average contig length (1184 vs 703) (Table 1). Moreover, 89.4% of the contigs from the study of Logacheva *et al.* [33] were covered in our assembled transcripts. Therefore, the assembled contigs in our study should provide a useful resource for future research on *F. tataricum*.

**Table 1:** **Comparison of Illumina sequencing data with reported 454 sequencing data**

	**Illumina sequencing**	**454 sequencing**
No. of reads	267,438,632	229,031
Average length of reads	100	341
Total nucleotides	26.7 billion	0.078 billion
No. of assembled contigs	39,815	25,401
Average length of contigs (Min-max)	1184 (201–25284)	703 (46–3298)
No. of reads per contig, mean	3008 (100)	7.5 (2–295)
(min-max)		

**Figure 3:**
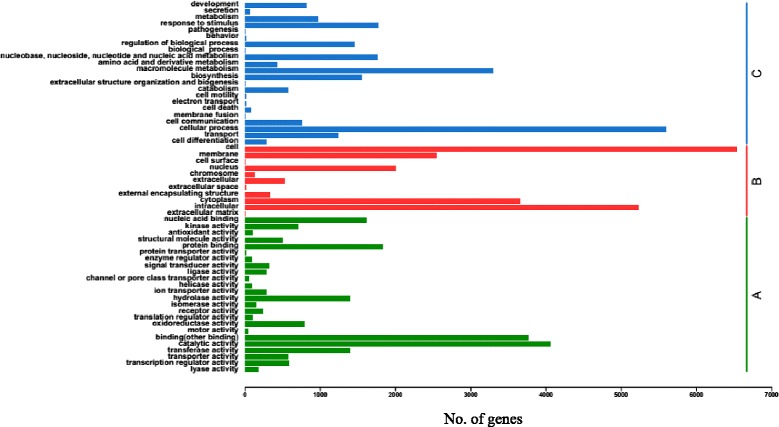
**Gene ontology (GO) analysis of selected genes.** A total of 8110 genes were categorized into three groups: Molecular function **(A)**, Cell component **(B)** and Biological process **(C)**.

BLAST searches revealed that 20,605 of 39,815 contigs had significant matches in the NCBI non-redundant protein database. Gene Ontology (GO) analysis of the matched contigs identified 8110 genes that were categorized into different GO groups (Figure 3). Some of the gene categories are partially redundant, which led to some genes being categorized into more than one group. In the molecular function category, genes assigned to the “catalytic activity” and “binding (other binding)” groups are highly enriched. In the cellular component category, genes in the “cell” and “intracellular” groups were the most abundant. In the biological process category, the “cellular process” and “macromolecule metabolism” groups contain the highest number of genes (Figure 3).

### Calculation and validation of RNA-seq expression data

**Figure 4:**
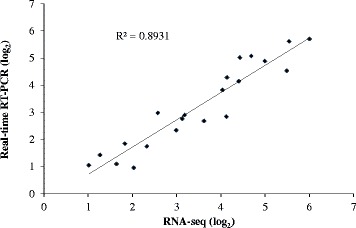
**Validation of the expression data from RNA-seq analysis via real-time RT-PCR analysis.** Fourteen genes exhibiting diverse expression profiles in the RNA-Seq data were chosen for real-time RT-PCR analysis. Average value of each RNA-seq expression data was plotted against that from quantitative real-time PCR and fit into a linear regression. Both x- and y-axes were shown in log_2_ scale.

The expression of each gene from the RNA-seq data was calculated by reads per kilobase of exons per million mapped reads. Although we used all 6 samples for the assembly of the transcripts, all genes identified had read coverage on each sample (data not shown), suggesting that our RNA sequencing of each sample was deep enough to allow expression analyses for all the genes. To verify the RNA-seq expression data, we selected 14 genes displaying diverse expression profiles in the root tips and/or basal roots for real-time RT-PCR analysis. A significant correlation (R^2^ = 0.89) was observed between two data sets (Figure 4). These results confirm the reliability of our RNA-seq expression data.

### Global effect of Al stress on gene expression

**Figure 5:**
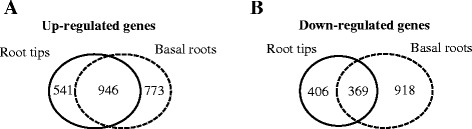
**Genes upregulated and downregulated in the root tips and basal roots after exposure to Al stress. (A)** Diagrams showing the genes upregulated by Al in the root tips (black circle) and basal roots (dotted circle). **(B)** Diagrams showing the genes downregulated by Al in the root tips and basal roots.

In the root tips, there are 1487 genes up-regulated and 775 genes down-regulated (|log_2_FC (fold change)| ≥ 1, FDR (false discovery rate) ≤ 0.001) under Al stress (Additional file 3: Table S3). Although root tips are known to be the main sites for Al detoxification, we found that there were also a large number of genes affected in the basal roots by Al stress, with 1719 genes being upregulated and 1287 genes being downregulated (Additional file 4: Table S4). GO enrichment analysis showed that the upregulated genes in both root tips and basal roots were significantly overrepresented in four categories: “Response to stimulus”, “Antioxidant activity”, “Extracellular” and “Cell death” (Table 2) (FDR ≤ 0.001), although the upregulated genes in the “Cell death” group were not significantly enriched in the basal roots due to the strict cut-off criteria used. This result suggests that defensive genes and genes encoding extracellular-localized proteins, such as cell wall components, were preferentially induced in expression by Al stress. The upregulated genes in the root tips and basal roots were also subjected to KEGG pathway enrichment analysis. Genes in two pathways, “Xenobiotics biodegradation and metabolism” and “Lipid metabolism”, were significantly enriched in both the root tip and basal root region (Table 3). The enrichment of genes in the lipid metabolism pathway supports the observation that Al can interfere with the function of the plasma membrane and induce its lipid peroxidation [34,35]. Together, these results suggest that Al toxicity can act on both the root tip and the basal root region and that both regions have evolved some common mechanisms of Al responsiveness in buckwheat. Further support for this statement came from the fact that a large portion of the upregulated or downregulated genes were shared between the root tips and the basal root region, with 946 and 369 genes being upregulated and downregulated in both root regions, respectively (Figure 5). By contrast, genes in “Carbohydrate metabolism” pathway were only significantly enriched in the root tip region, and genes in four pathways, “signal transduction”, “Environmental adaptation”, “Immune system” and “Sensory system” were overrepresented in the basal roots but not in the root tips under Al stress (Table 3). Expression analysis also showed that some genes were upregulated or downregulated only in the root tips or the basal roots (Figure 5; Additional file 3: Table S3, Additional file 4: Table S4). Therefore, the root tip and basal root region may also possess different mechanisms of Al responsiveness in buckwheat.

**Table 2:** **Gene ontology enrichment analysis of upregulated genes in root tips and basal roots exposed to Al stress**

	**No. of genes in the whole transcriptome**	**Root tip region**		**Basal root region**	
		**No. of upregulated genes**	**FDR**	**No. of upregulated genes**	**FDR**
Response to stimulus	1772 (8110)	165 (484)	3.20E-09	193 (526)	1.67E-14
Antioxidant activity	98 (8110)	19 (484)	2.77E-05	67 (526)	5.96E-07
Extracellular	528 (8110)	57 (484)	6.03E-05	18 (526)	2.57E-04
Cell death	74 (8110)	14 (484)	3.45E-04	13 (526)	3.42E-03

**Table 3:** **KEGG enrichment analysis of upregulated genes in root tips and basal roots exposed to Al stress**

**KEGG pathway**	**No. of genes in the whole transcriptome**	**Root tip region**		**Basal root region**	
		**No. of upregulated genes**	**FDR**	**No. of upregulated genes**	**FDR**
Xenobiotics biodegradation and metabolism	188 (3282)	28 (162)	4.67E-07	28 (180)	1.34E-06
Lipid metabolism	307 (3282)	30 (162)	7.19E-04	31 (180)	7.81E-04
Carbohydrate metabolism	710 (3282)	55 (162)	7.19E-04	44 (180)	2.33E-01
Signal transduction	669 (3282)	45 (162)	3.02E-02	65 (180)	1.34E-06
Environmental adaptation	144 (3282)	15 (162)	1.26E-02	22 (180)	1.25E-05
Immune system	251 (3282)	10 (162)	9.54E-01	31 (180)	1.64E-05
Sensory system	23 (3282)	2 (162)	2.25E-01	6 (180)	6.82E-04

### Effect of Al on the expression of organic acid metabolism and secretion-related genes

**Figure 6:**
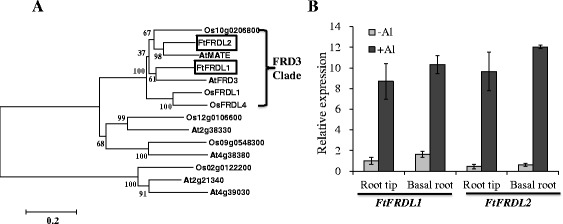
**Phylogenetic and expression analysis of FRD3 homologs in buckwheat. (A)** Phylogenetic tree of buckwheat FRD3 homologs (boxed FtFRDL1 and FtFRDL2) and other MATE homologs from Arabidopsis and rice. **(B)** Effect of Al stress on the expression of *FtFRDL1* and *FtFRDL2* in different root regions. The data were normalized to *FtFRDL1* expression in the root tips without Al treatment. Data shown are means ± SD (n = 3).

Secretion of oxalate from the root tips in response to Al and chelation of Al by oxalate within root cells are well-characterized mechanisms of Al tolerance in buckwheat [14,28,36,37]. We therefore investigated the effect of Al on the expression of genes involved in organic acid synthesis or metabolism. The results showed that the expression of genes putatively involved in the tricarboxylic acid cycle, including key enzymes such as malate dehydrogenase and citrate synthase, was not induced by Al stress (Additional file 5: Figure S1), which is consistent with evidence that organic acid metabolism is not a rate-limiting step for Al-induced release of organic acids [38-40]. Interestingly, we found that two genes belonging to the MATE (*M*ultidrug *A*nd *T*oxic compound *E*xtrusion) family were induced in expression in both the root tips and basal roots by Al stress (Figure 6). Phylogenetic analysis indicated that the two MATE members, FtFRDL1 and FtFRDL2, clustered with the citrate transporter AtFRD3, a founding member of the FRD3 subfamily (Figure 6A). Although the basal expression of *FtFRDL1* in the absence of Al was higher than that of *FtFRDL2*, the latter was more induced by Al, resulting in a similar expression level of the two genes after exposure to the Al stress (Figure 6B). The MATE genes from the FRD3 clade have been shown to be involved in transporting citrate [18,19,41,42]. Although Al-activated citrate secretion is not the Al-tolerance mechanism in buckwheat, citrate might be transported into the xylem for Al chelation and translocation [29]. Therefore, it is possible that the two MATE genes are involved in the release of citrate into the xylem.

### Expression and phylogenetic analysis of Al-tolerance gene homologs

**Figure 7:**
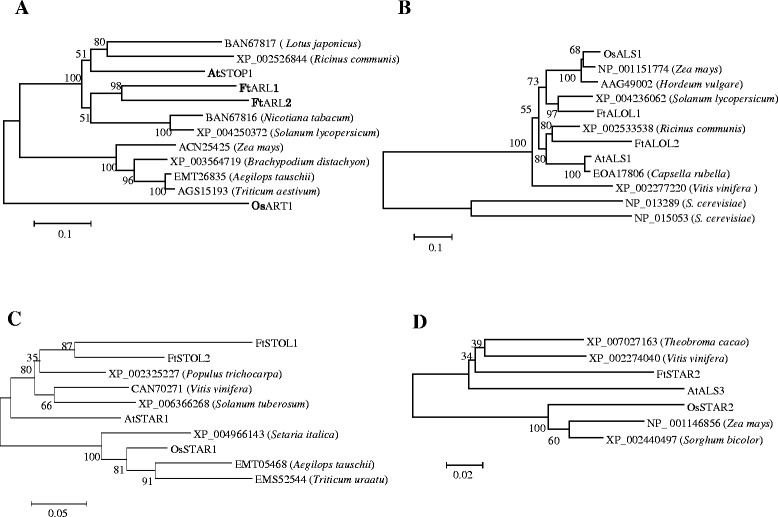
**Phylogenetic analysis of homologs of ART1/STOP1 (A), ALS1 (B), STAR1 (C) and STAR2/ALS3 (D) in different species.** Accession numbers and species names are shown in the tree except those homologs from Arabidopsis and rice.

**Figure 8:**
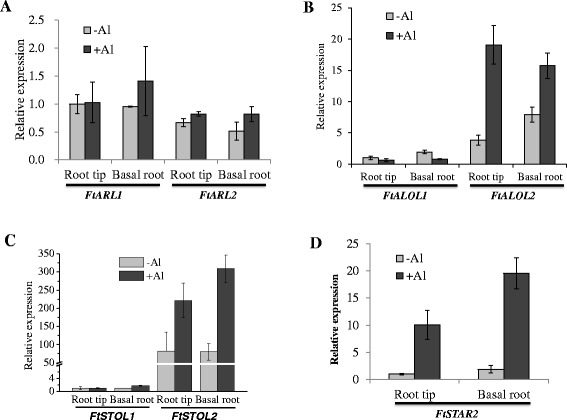
**Expression analysis of Al-tolerance gene homologs in different root regions under different Al conditions. (A)** *ART1* homologs, *FtARL1* and *FtARL2*. **(B)** *ALS1* homologs, *FtALOL1* and *FtALOL2*. **(C)** *STAR1* homologs, *FtSTOL1* and *FtSTOL2*. **(D)** *FtSTAR2*. The data were normalized to the expression of gene homolog1 in the root tips without Al treatment. Data shown are means ± SD (n = 3).

A number of genes required for Al tolerance in rice and Arabidopsis have been cloned and characterized recently. To understand the mechanisms of Al tolerance in the Al hyperaccumulator buckwheat, we performed expression and phylogenetic analysis of homologs of four conserved Al-tolerance genes in rice and Arabidopsis, ART1/STOP1, ALS1, STAR1 and STAR2/ALS3. We identified two homologs of ART1, namely ARL1 and ARL2 (*A*RT1-*L*ike) in buckwheat. Phylogenetic analysis indicated that both ARL1 and ARL2 are closer to Arabidopsis STOP1 than to rice ART1 (Figure 7A), suggesting that the duplication event of ART1 in buckwheat happened after the dicot-monocot split. Real-time RT-PCR analysis showed that both *ARL1* and *ARL2* were equally expressed in the root tips and basal roots, and their expression was not affected by the Al treatment (Figure 8A).

There were two ALS1 homologs found in buckwheat. Interestingly, one of the ALS1 homologs (FtALOL1, *AL*S *O*ne-*L*ike 1) is closer to rice OsALS1, whereas the other (FtALOL2) is closer to Arabidopsis AtALS1 (Figure 7B), suggesting that ALS1 duplication in buckwheat occurred before the split of monocots and dicots. Expression analysis showed that *FtALOL2* transcript accumulation was higher than that of *FtALOL1* in the roots, and that *FtALOL2* expression was induced by the Al stress, to a greater extent in the root tips compared with the basal roots (Figure 8B). By contrast, the expression of *FtALOL1* was downregulated by the Al treatment.

We identified two homologs of STAR1, STOL1 and STOL2 (*ST*AR-*O*ne *L*ike), in buckwheat. Both STOL1 and STOL2 fall into the dicot group (Figure 7C), suggesting that STAR1 was duplicated in buckwheat after the evolutionary divergence of dicots and monocots. Quantitative RT-PCR analysis showed that the expression level of *STOL2* was more than 50 fold higher than that of *STOL1* in the roots (Figure 8C), and the expression of *STOL2* was induced in both the root tips and the basal root region by Al stress, but that of *STOL1* was not. These results suggest that STOL2 may play a major role for Al tolerance in buckwheat roots. Whether STOL1 plays an important role in the shoots requires further investigation. In contrast to STAR1, there was only one homolog of STAR2 in buckwheat (Figure 7D). The expression of *FtSTAR2* in both the root tips and the basal root region was highly induced by Al stress (Figure 8D).

## Discussion

Similar to common buckwheat, tartary buckwheat was able to accumulate high levels of Al in the roots and shoots in a short-term hydroponic experiment (Figure 1). This result is consistent with a recent report showing that tartary buckwheat shares similar mechanisms of Al detoxification and accumulation with common buckwheat species [30].

Through Illumina high-throughput mRNA sequencing and *de novo* assembly of the transcripts with an optimized method, we constructed nearly 40,000 transcript contigs of high quality in tartary buckwheat (*F. tataricum*). Compared with previous 454 sequencing in *F. tataricum* [33], our high-throughput mRNA sequencing generated 300 fold more nucleotides and therefore enabled us to assemble more contigs and obtain longer transcripts (Table 1). When we cloned full transcripts of the gene homologs of *FRD3*, *ART1*, *ALS1*, *STAR1* and *STAR2* by 5′-RACE and 3′-RACE PCR in buckwheat, we found that in fact all the homologs had full length open reading frames (ORFs) in our assembled contigs, whereas the ORFs of the homologs from previous 454 sequencing data were incomplete (Data not shown). Thus, our assembled transcripts provide a platform for future research on buckwheat.

Our differential expression analysis of RNA-seq data revealed that a large number of genes upregulated or downregulated by Al stress were shared in the root tips and basal roots (Figure 5), which suggested that at the cellular level, Al toxicity might not be restricted to the root tip cells, but can also act on the basal root cells. This result is consistent with a previous report that Al could affect the expression of some genes in both the root tip and basal root region of rice [43]. GO and KEGG enrichment analysis revealed that genes categorized into “Response to stimulus”, “Antioxidant activity” and “Lipid metabolism” were preferentially induced in expression by Al stress (Tables 2 and 3), which supported previous observations that Al can induce the peroxidation of lipids and the production of reactive oxygen species (ROS), and that plant roots are able to increase the expression of antioxidant genes such as glutathione S-transferase (*GST*) genes to cope with Al toxicity [34,44,45]. Additionally, the expression of genes categorized as “Extracellular” or putatively involved in “Carbohydrate metabolism” were also increased in the root tips in response to Al, which was consistent with the concept that the root cell wall is the primary target site of Al toxicity [2,44].

Both common and tartary Buckwheat are able to secrete oxalate to chelate and detoxify Al in the rhizosphere [13,14,30], although the genes responsible for the release of oxalate from the roots have not been identified. There are two temporal patterns adopted by plants for Al-activated organic acid release [27]. In Pattern I, exudation of organic acids is rapidly activated by Al exposure and there is no discernible delay observed between the addition of Al and the onset of organic acid anion release, whereas in Pattern II the secretion of organic acids is delayed for several hours after exposure to Al. The secretion of oxalate in buckwheat is rapid and at a constant level after the exposure to Al [13,14], consistent with a Pattern I response. Recent reports on wheat *ALMT1* and barley *HvAACT1* indicate that the expression of genes encoding transporters for the secretion of organic acid in Pattern I is constitutive and not responsive to Al stress [17,18]. Therefore, it will be difficult to use RNA-seq analysis to identify the genes responsible for the exudation of oxalate in buckwheat since their expression might not be affected by Al stress. An alternative approach could be to screen mutants defective in oxalate secretion, followed by cloning of the responsive genes through map-based cloning techniques, to isolate genes encoding oxalate transporters.

Interestingly, we found that the expression of two homologs of *FRD3* was highly induced by the Al treatment (Figure 6B). The MATE genes in the *FRD3* subgroup have been demonstrated to be involved in the translocation of iron through the release of citrate to the xylem or in Al tolerance through citrate release to the rhizosphere in Arabidopsis [41,42]. Although buckwheat secretes oxalate instead of citrate to the rhizosphere for the detoxification of Al, it is possible that the plant may release citrate to the xylem for the translocation of Al because the Al-citrate complex is the predominant form of Al in the xylem [29], which could be mediated by the FRD3-like transporters in buckwheat. Similarly, release of citrate into the xylem is required for iron translocation in both dicot and monocot species [41,46]. The requirement for citrate in the xylem translocation of both iron and Al in buckwheat would need to be coordinated closely. Because buckwheat hyperaccumulates Al in the shoots, the amount of citrate required for Al translocation in the xylem could be substantial. In the presence of Al, the amount of citrate release to the xylem would have to be increased, triggering the induction of genes involved in citrate release. The increased expression of the two *FRD3* homologous genes under Al treatment supports our speculation. In the future, it will be critical to determine whether the two genes are involved in the translocation of Al and/or iron in buckwheat and to examine how Al activates the expression of the two genes.

The requirement of ART1/STOP1, ALS1, STAR1 and STAR2/ALS3 for Al tolerance appears to be conserved and ubiquitous in monocot and dicot species, and they do not have close homologs in the rice and Arabidopsis genomes [20-26]. By contrast, we found that three of the four genes had been duplicated in buckwheat (Figure 7). The two homologs of *ART1*, a putative zinc-finger transcription factor, were expressed at a similar level and largely unaffected by the Al stress (Figure 8A), similar to the expression pattern of *ART1* and *STOP1*. It remains to be demonstrated whether the two homologs are redundant or have different tissue-specific expression patterns. It will be also interesting to investigate whether the two *ART1* homologs are required for Al translocation and accumulation in the shoots of buckwheat. In contrast to the *ART1* homologs, the two homologs of *STAR1*, a putative ABC transporter, displayed an unequal expression pattern, with *FtSTOL2* accumulating to a higher level than *FtSTOL1* in the roots (Figure 8C). Furthermore, the expression of *FtSTOL2* was highly induced by the Al treatment, whereas that of *FtSTOL1* was unaffected. These results suggest that *FtSTOL2* is the major gene required for Al tolerance in the roots of buckwheat. Although FtSTOL2 had greater sequence similarity to Arabidopsis AtSTAR1 than to rice STAR1 (Figure 7C), the expression pattern of *FtSTOL2* was similar to rice *STAR1*. Arabidopsis *AtSTAR1* is mainly expressed in the root tip region and is not responsive to Al stress [23], whereas both buckwheat *FtSTOL2* and rice *STAR1* were equally expressed in both the root tip and basal root region and their expression was highly induced by Al [22]. Unlike STAR1 homologs, there was only one homolog of STAR2 in buckwheat. The expression of *FtSTAR2* was also greatly increased after exposure to Al stress (Figure 8D), which reinforced the view that Al-induced expression of *STAR2* is a conserved mechanism in plants since previous reports also showed that rice *STAR2* and Arabidopsis *ALS3* were increased in expression after exposure to Al [22,24].

Whereas the duplication of ART1/STOP1 and STAR1 appears to occur after the divergence of dicots and monocots, ALS1 duplication may have occurred before the split of monocots and dicots (Figure 7). In fact, duplication of ALS1 appears to be an ancient event because the yeast *Saccharomyces cerevisiae* has two copies of ALS1 in its genome (Figure 7B). While many plants appear to have lost one copy of ALS1, tartary buckwheat retains both copies. We also found that tea (*Camellia sinensis*) has two copies of ALS1 (Unpublished data). As both buckwheat and tea are Al hyperaccumulators and highly tolerant to Al stress, these results suggest that retaining two ALS1 copies might be a common feature for Al hyperaccumulators with both homologs playing important roles in the tolerance and/or distribution of Al. In addition, although phylogenetic analysis showed that FtALOL2 was closer to Arabidopsis AtALS1 (Figure 7B), the expression pattern of *FtALOL2* was more like that of rice *OsALS1* (Figure 8B). *AtALS1* was preferentially expressed in the root tip region and its expression was not affected by the Al treatment [25], whereas both *FtALOL2* and *OsALS1* had greater expression in the basal roots than in the root tips, and their expression was induced by Al in both root regions [26]. Conversely, *FtALOL1* expression was not induced by Al stress even though FtALOL1 had greater sequence similarity to the monocot OsALS1 (which is upregulated by Al stress) than to the dicot AtALS1 (Figures 7B and 8B). In the future, it will be essential to determine the in vivo function of the two ALS1 homologs in buckwheat and to examine whether they have redundant functions in Al tolerance and/or accumulation in roots and shoots of buckwheat.

Compared with the Al-sensitive species Arabidopsis, the Al-tolerance species rice is able to express high levels of the conserved Al-tolerance genes in the presence of Al to overcome Al stress. Similar to rice, tartary buckwheat also showed high expression of the Al-tolerance gene homologs under Al stress, although the Al-tolerance species buckwheat is evolutionarily closer to Arabidopsis than rice (Figures 7 and 8). These suggest that buckwheat has evolved high expression of Al-tolerance genes to detoxify Al. In addition, buckwheat has experienced gene duplication of *ART1/STOP1*, *STAR1* and *ALS1*. Since buckwheat can accumulate high levels of Al in addition to having high tolerance to Al, gene duplication might be important for buckwheat to coordinate the Al tolerance and Al accumulation in roots and shoots. In this regard, it is interesting to note that zinc/cadmium hyperaccumulation in *Arabidopsis halleri* also involves duplication of key genes responsible for metal translocation and detoxification [47]. Further functional analysis by creating knock-down or knock-out mutants will be required to reveal the role of each homologous gene in Al detoxification and accumulation in buckwheat.

## Conclusions

Through genome-wide mRNA sequencing analysis, we constructed about 40,000 high-quality transcripts in tartary buckwheat, which provide a sequence basis for further investigation into the molecular mechanisms of Al tolerance and accumulation in buckwheat. Our RNA-seq analysis reveals that the root tip and the basal root region of tartary buckwheat may possess both common and different mechanisms of Al responsiveness and that organic acid metabolism is not the rate-limiting step for organic acid secretion induced by Al in buckwheat. We propose that xylem loading of Al may be a rate-limiting step for the translocation of Al from roots to shoots in buckwheat and that two putative citrate transporters, FtFRDL1 and FtFRDL2, may be required for the translocation of Al via the release of citrate into the xylem for complexation with Al. We also propose that buckwheat has experienced duplication and subfunctionlization of key genes to coordinate the Al tolerance and Al accumulation.

## Methods

### Plant materials and growth conditions

Wild-type buckwheat used for transcriptome analysis was *Fagopyrum tataricum* (cv. Xiqiao2). The Xiqiao2 variety is widely cultivated in Liangshan prefecture of Sichuan province in China and we collected its seeds at a food market in that area. Seeds were soaked in deionized water for 6 h in the dark at room temperature and then transferred to nets floating on a 0.5 mM CaCl_2_ solution in a 3-liter plastic container. The solution was renewed every day. Plants were grown in a growth chamber at 23°C in the dark. Three days later, the seedlings were pretreated with a 0.5 mM CaCl_2_ solution at pH 4.5 for 24 h before being exposed to a 0.5 mM CaCl_2_ solution containing 0 or 50 μM AlCl_3_ at pH 4.5 for 6 h. Root tips (0–2 cm) and basal roots (2-4 cm) with three biological replicates were sampled in both –Al and + Al conditions for RNA-seq. For each sample, 40–50 root segments were collected and frozen in liquid nitrogen within 5 min for RNA isolation. Due to the cost consideration, RNA-seq was performed on two replicates of root tips and one replicate of basal roots in both –Al and + Al treatments. For real-time RT-PCR analysis, all the three replicates were used to quantify the gene expression. It has been shown that the reliability of differential expression in RNA-seq is dependent on the sequencing depth [48]. To ensure reliability, our samples were sequenced to around 250 fold coverage of each contig on average (Additional file 2: Table S2). Furthermore, the RNA-seq data were verified by quantitative RT-PCR (Figure 4).

### Determination of Al accumulation

For determination of Al concentrations in roots and shoots, two-week-old seedlings of tartary buckwheat (cv. Xiqiao2) and common buckwheat (cv. Jiangxi) were exposed to a 0.5 mM CaCl_2_ solution containing 0, 10, 20 or 50 μM AlCl_3_ for 24 h and then to one-fifth strength Hoagland’s solution for another 24 h. After intermittent Al treatment for 8 d, roots and shoots were sampled for the determination of Al concentrations. The samples were dried at 60°C in an oven for a week and digested with HNO_3_. The Al concentration was measured by Inductively Coupled Plasma Mass Spectrometry (Nexion 300X ICP-MS, Perkin Elmer, USA).

### RNA isolation, library construction and Illumina deep sequencing

Total RNA was extracted using General Plant RNA Extraction Kit (BioTeke, China). The extracted RNA was digested with DNase I (TAKARA) to remove contaminated DNA. mRNAs were purified from the total RNA using Dynabeads Oligo (dT)_25_ (Life Technologies). The derived mRNAs were fragmented and reverse transcribed into first-strand cDNAs with random hexamer and then the second-strand cDNAs were synthesized by using a NEBNext UltraTM RNA Library Prep Kit for Illumina (NEB). The double-stranded cDNAs were purified and ligated to adaptors for Illumina paired-end sequencing. The cDNA library was sequenced using the Illumina HiSeq2500 system by Shanghai Hanyu Biotech lab (Shanghai, China).

### Transcriptome assembly and annotation

Raw reads were filtered using the FastaX package to remove adaptor sequences and low-quality reads (base quality < 20, read length < 40 bp). The obtained clean reads of all six samples were assembled using the Trinity program [32] with the following parameters: k-mer = 25; minimum k-mer coverage = 2; maximum length expected between fragment pairs = 500; minimum overlap of reads with growing transcript PE = 75; maximum number of reads to anchor within a single graph = 200,000. Putative coding sequences of the assembled transcripts were predicted by “get orf” in the EMBOSS package. To annotate the assembled transcripts, BLASTp searches (e-value < 0.00001) were performed among all-predicted coding sequences and protein databases including NCBI, Swiss-Prot, KEGG and COG. Functional annotation using gene ontology terms (GO; http://www.geneontology.org) was analyzed using the BLASTp algorithm against the Swiss Prot database by the GoPipe program of gene2go software at ftp://203.110.175.109. COG/KOG and KEGG pathways annotation was carried out using Blastall software against the Cluster of Orthologous Groups database and Kyoto Encyclopedia of Genes and Genomes database, respectively.

### Differential expression analysis and GO and KEGG enrichment analysis

The cleaned reads of each sample were mapped back to assembled contigs by bowtie2 with the following parameters: maximum mismatches in seed alignment = 1; length of seed substrings = 22. The assembled contigs with more than 10 reads mapped were subjected to differential expression analysis. For the expression analysis, the number of clean reads for each contig was calculated and then normalized to Reads Per Kb per Million reads (RPKM). The expression difference of each contig between different treatments was calculated based on the MARS model (MA-plot-based method with Random Sampling model) using the DEGseq package. FDR (false discovery rate) value less than 0.001 and |log_2_(fold change)| ≥ 1 were used as the threshold to judge the significance of gene expression difference.

Differentially expressed genes were extracted for GO functional enrichment analysis and KEGG pathway enrichment analysis. The enrichment analysis was tested using a hypergeometric test at a significance cutoff of ~0.1% false discovery rate (FDR).

### Real-time RT-PCR

One microgram of total RNA was used to synthesize the first-strand cDNAs by using HiScript® 1st Strand cDNA Synthesis Kit (Vazyme). One twentieth of the cDNA products and the SYBR® Green Master Mix kit (Vazyme) were used for real-time RT-PCR analysis. The *SAND* gene was used as the internal control, which has been shown to be one of most stable reference genes in buckwheat [49]. Primers for real-time RT-PCR analysis are listed in Additional file 6: Table S5. Data were collected in accordance with the CFX96 Real-Time PCR Detection System (Bio-Rad).

### Phylogenetic analysis

Phylogenetic analysis was carried out by using the MEGA4.0 program (http://www.megasoftware.net). The neighbor-joining method was used to construct the phylogenetic tree with 1000 bootstrap trials by MEGA4.0 [50].

### Availability of supporting data

Illumina high throughput mRNA sequencing data of *Fagopyrum tataricum* (cv. Xiqiao2) were deposited in the NCBI SRA database under following accession numbers: SRR1460477 and SRR1552100 (two replicates, root tip region, −Al condition), SRR1552203 and SRR1552215 (two replicates, root tip region, +Al condition), SRR1552101 (basal root region, −Al condition), and SRR1552217 (basal root region, +Al condition). The phylogenetic trees were deposited in treebase (http://treebase.org) under following URL: http://purl.org/phylo/treebase/phylows/study/TB2:S16751?x-access-code=3031f75cf503d89e82b52c6ba8769d97&format=html. The data sets supporting the results of this article are included within the article and its additional files.

## Additional files

Additional file 1: Table S1. Summary of the Illumina mRNA sequencing. Additional file 2: Table S2. The sequences and annotation of assembled transcript contigs in *Fagopyrum tataricum*. Additional file 3: Table S3. Genes upregulated and downregulated in root tips after exposure to Al for 6 h. Additional file 4: Table S4. Genes upregulated and downregulated in basal roots after exposure to Al for 6 h. Additional file 5: Figure S1. Effect of Al stress on the expression of genes putatively involved in the tricarboxylic acid cycle. The putative genes for each enzyme were indicated with the name “Comp…”. The values on the right side of the genes indicated fold changes of each gene expression in the root tips under Al stress, which are calculated from the RNA-seq data. Additional file 6: Table S5. Primers used for quantitative RT-PCR analysis.

## Abbreviations

Al: Aluminum
GO: Gene Ontology
KEGG: Kyoto Encyclopedia of Genes and Genomes
MATE: Multidrug and toxic compound extrusion
ORF: Open reading frame
RACE: Rapid-amplification of cDNA ends
RNA-seq: High-throughput RNA sequencing
ROS: Reactive oxygen species
