# Supplementary figures and images for: Genome-wide transcriptomic and phylogenetic analyses reveal distinct aluminum-tolerance mechanisms in the aluminum-accumulating species buckwheat (Fagopyrum tataricum)
Source: BMC Plant Biol. 2015 Jan 21;15:16. doi: 10.1186/s12870-014-0395-z (PMC4307214; doi:10.1186/s12870-014-0395-z)

## Slide 1
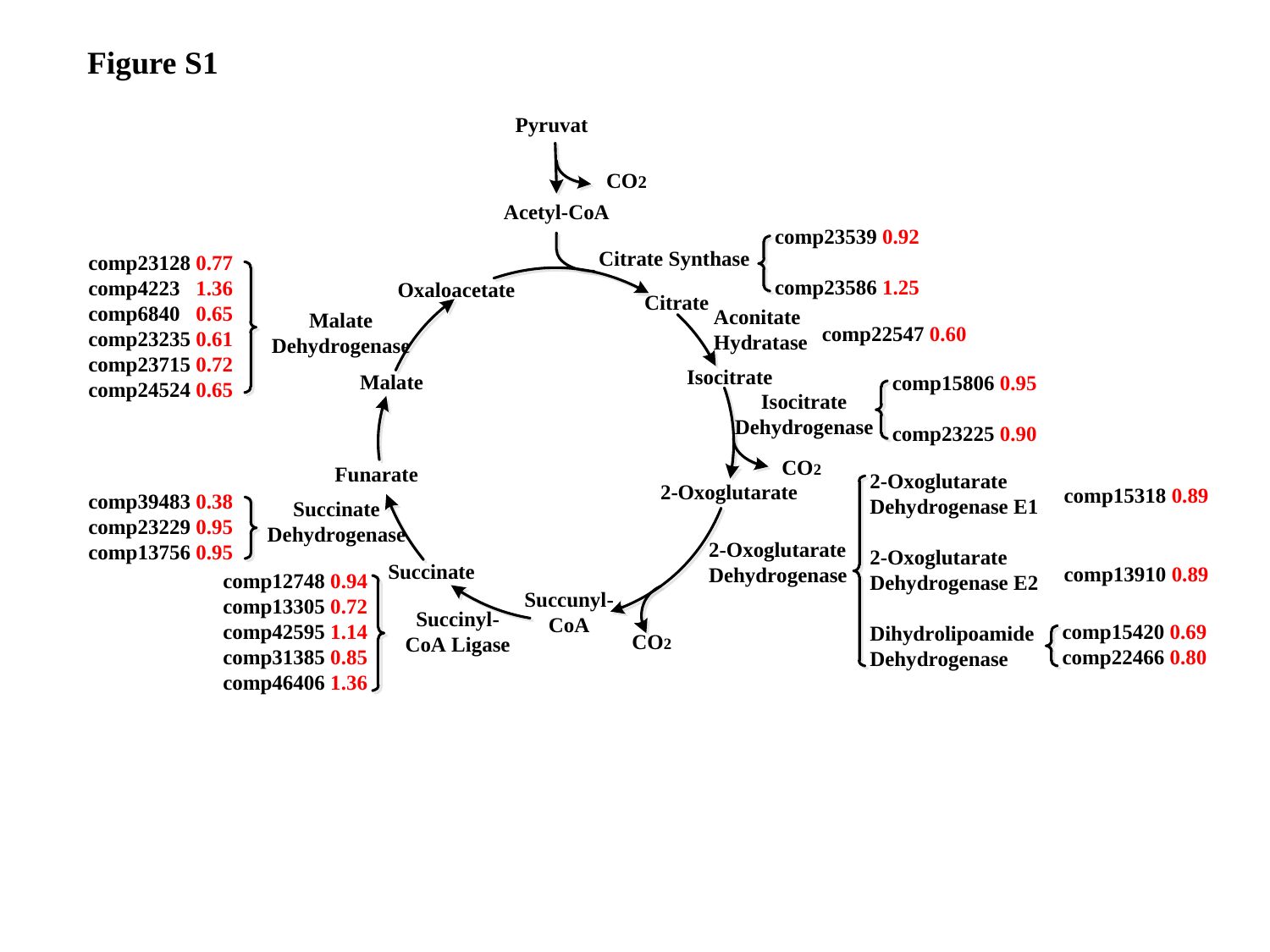

Figure S1

Supplement: Additional file 5: Figure S1. — Effect of Al stress on the expression of genes putatively involved in the tricarboxylic acid cycle. The putative genes for each enzyme were indicated with the name “Comp…”. The values on the right side of the genes indicated fold changes of each gene expression in the root tips under Al stress, which are calculated from the RNA-seq data. [file 12870_2014_395_MOESM5_ESM.pptx]
